# Core outcome sets in cancer and their approaches to identifying and selecting patient-reported outcome measures: a systematic review

**DOI:** 10.1186/s41687-020-00244-3

**Published:** 2020-09-15

**Authors:** Imogen Ramsey, Marion Eckert, Amanda D. Hutchinson, Julie Marker, Nadia Corsini

**Affiliations:** 1 Rosemary Bryant AO Research Centre, UniSA Clinical & Health Sciences, University of South Australia, Adelaide, Australia; 2 UniSA Justice & Society, University of South Australia, Adelaide, Australia; 3 Cancer Voices South Australia, Adelaide, Australia

**Keywords:** Cancer, Core outcome set, Patient-reported outcomes

## Abstract

**Objectives:**

Issues arising from a lack of outcome standardisation in health research may be addressed by the use of core outcome sets (COS), which represent agreed-upon recommendations regarding what outcomes should be measured as a minimum in studies of a health condition. This review investigated the scope, outcomes, and development methods of consensus-based COS for cancer, and their approaches and criteria for selecting instruments to assess core patient-reported outcomes (PROs).

**Methods:**

Studies that used a consensus-driven approach to develop a COS containing PROs, for use in research with cancer populations, were sought via MEDLINE, CINAHL, Embase, Cochrane Library, and grey literature.

**Results:**

Seventeen studies met the inclusion criteria. Most COS (82%) were specific to a cancer type (prostate, esophageal, head and neck, pancreatic, breast, ovarian, lung, or colorectal) and not specific to an intervention or treatment (76%). Conducting a systematic review was the most common approach to identifying outcomes (88%) and administering a Delphi survey was the most common approach to prioritising outcomes (71%). The included COS contained 90 PROs, of which the most common were physical function, sexual (dys) function, pain, fatigue, and emotional function. Most studies (59%) did not address how to assess the core PROs included in a set, while 7 studies (41%) recommended specific instruments. Their approaches to instrument appraisal and selection varied.

**Conclusion:**

Efforts to standardise outcome assessment via the development of COS may be undermined by a lack of recommendations on how to measure core PROs. To optimise COS usefulness and adoption, valid and reliable instruments for the assessment of core PROs should be recommended with the aid of resources designed to facilitate this process.

## Background

Appropriate outcome selection, measurement, and reporting are crucial to the design of quality research studies [1]. For research to inform practice and policy, the outcomes measured need to be considered meaningful and relevant by patients, clinicians, and other health service users and stakeholders [2]. There is increasing recognition that a lack of standardisation in the outcomes examined in clinical trials is problematic. When outcome selection is at the discretion of the research team, it is possible that poorly defined or irrelevant outcomes may be selected, or that study findings may be selectively reported leading to an exaggeration of treatment effects [3]. Furthermore, inconsistency in the outcomes assessed across studies can prevent data comparison and synthesis [3, 4].

Concerns about outcome standardisation have prompted efforts to identify minimum requirements for outcome measurement and reporting, known as core outcome sets (COS) [1]. A COS is an agreed-upon set of recommendations stipulating what should be measured and reported as a minimum in research on a health condition, including but not limited to comparative effectiveness trials [2, 5]. Interest in standardising outcome measurement was championed by the OMERACT (Outcome Measures in Rheumatology) consensus initiative [6], which has endorsed the use of consensus-based core outcome sets in rheumatology since 1992 and published a framework and recommended process for core outcome set development [7]. Recent interest in outcome standardisation has led to a proliferation of COS for various diseases and interventions, and across health care sectors including research, performance assessment, and quality improvement. Consequently, the Core Outcome Measures in Effectiveness Trials (COMET) initiative [8] was established to convene core outcome set developers across disciplines and provide methodological guidance to support robust COS development [1]. Members of the COMET and OMERACT teams led the development of standards for COS study design (COS-STAD) [9], protocol items (COS-STAP) [10], and reporting (COS-STAR) [9], and guidelines for selecting instruments for a COS (the latter in collaboration with the Consensus-based Standards for the Selection of Measurement Instruments (COSMIN) initiative) [11]. All initiatives agree that the development of a core outcome set first requires consensus to be reached among key stakeholders on “what” to measure (i.e., the core outcomes) and then on “how” to measure the outcomes (i.e., the instruments) [11]. While they all recognise the importance of determining how to measure outcomes once a COS is defined (i.e., which instrument should be used), treating this as an ancillary process may undermine COS efforts. Use of different instruments to measure a given outcome remains a major challenge impeding the usefulness of research involving patient-reported outcomes (PROs) [3].

A PRO is any report of a patients’ health status provided directly by the patient, typically via validated instruments known as patient-reported outcome measures (PROMs) [12]. An extensive range of PROMs have been developed for different purposes (e.g., screening, clinical management, surveillance), within different disciplines (e.g., psychology, oncology, physiotherapy) and for different populations (e.g., general, disease-specific) [3]. They may assess one or multiple domains, contain subscales and/or single items, and vary in content, terminology, and scoring. The problems arising from inconsistent use of PROMs were highlighted in a systematic review of cancer survivorship registries, which found substantial variation in the PROMs used by the registries and limited information about how or why particular outcomes and measures were selected [13]. For example, although six registries assessed ‘health status’, each used a different PROM. Some of these were generic preference-based (i.e., utility) instruments for the assessment of general health, others were for the assessment of health-related quality of life (HRQOL) among cancer populations, and one was intended specifically for long-term follow-up of cancer survivors [13]. The resulting differences in the outcome domains, terminology, subscales, and scoring prevented comparison and synthesis of data from the registries, which all sought to monitor the long-term wellbeing of cancer survivors [13].

For COS to be successfully implemented and adopted, researchers need to be able to measure the outcomes in a valid and consistent manner [14]. The COSMIN/COMET guideline provides methods to undertake this process in four steps: 1) conceptual considerations, 2) finding existing instruments by means of a literature review or search, 3) quality assessment of identified instruments based on psychometric properties and feasibility, and 4) recommendations on the selection of instruments for outcomes included in a COS [11]. While review findings indicate that methodology for developing COS has advanced in recent years [15, 16], methodology for selecting outcome measurement instruments for COS has not been critically examined. Given the expanding number of cancer-related COS containing PROs, an investigation of their characteristics and approaches to determining how to measure core PROs is warranted.

This review aimed firstly to identify and comprehensively describe all COS containing PROs that have been determined via consensus methods for use in research with cancer populations, and to examine what PROs they include. Secondly, the review aimed to identify what (if any) PROMs were recommended for assessment of the core outcomes and how the PROMs were selected, in order to identify where further guidance for the development of consensus-based COS may be required.

## Methods

A protocol was registered with the International Prospective Register of Systematic Reviews (PROSPERO). The review was conducted in accordance with the Preferred Reporting Items of Systematic Reviews and Meta-Analyses (PRISMA) guidelines (see Table S1 for the PRISMA Checklist).

### Inclusion and exclusion criteria

Studies were eligible if they had developed recommendations regarding what PROs should be measured as a minimum in health research involving adult populations with cancer using a consensus-driven methodology. Studies with consensus-based approaches were sought in order to focus on COS that had been developed since the release of resources such as the COMET Handbook 1.0 [1], COS-STAD checklist [17], OMERACT Handbook and Filter 2.0 [7], and COSMIN/COMET guideline [11], which all emphasise the consensus component of COS development.

As this review sought evidence regarding the selection of PROs and PROMs in studies that had determined consensus on a COS, studies that did not report findings from the development of a COS were excluded. This included study protocols, reviews of outcomes or outcome measurement instruments, conference or meeting proceedings, studies validating or evaluating a COS, and studies discussing general recommendations for outcome measurement or trial design. Due to the focus on PROs, studies that exclusively defined or standardised clinician-reported outcomes, clinical endpoints or terminology/criteria for diagnosis, staging, or response were excluded.

### Identification of studies

Searches were undertaken in October 2018 for studies describing the development of cancer-related COS using a consensus-driven methodology. The search combined MeSH words and terms for cancer, core outcome sets, and consensus approaches and was constructed in MEDLINE (via Ovid) then adapted for CINAHL, Embase, and Cochrane Library. The search strategy is documented in Table 1. The COMET database was also searched for entries listed under the disease category “cancer”. Grey literature was sourced by executing a simplified version of the database search in Google Scholar (Advanced).

**Table 1:** Search strategy for MEDLINE

Search #	Terms
1	exp Neoplasms/ or (cancer*or tumor* or tumour* or neoplas*).mp.
2	((set or sets or dataset? or “data set?” or outcome?) adj3 (core or minimum or standard*)).mp.
3	(standard* adj3 (terminology or reporting or recommend* or criteria or guideline?)).mp.
4	((endpoint? or “end point?”) adj3 (develop* or define? or defini* or establish* or determin* or specif* or recommend*)).mp.
5	2 or 3 or 4
6	Consensus/ or Delphi Technique/
7	(consensus or agree* or Delphi or meeting or “expert? panel?” or (group adj6 recommendation?)).mp.
8	6 or 7
9	1 and 5 and 8

### Study selection

Search results were pooled and duplicates removed. One reviewer screened the titles and abstracts of all citations identified in the searches for relevance and independent checks were performed by a second reviewer. The full texts of potentially relevant articles were obtained and assessed by one reviewer against the inclusion criteria and reasons for exclusion were documented. Ten percent of the excluded full texts were checked for correct exclusion by a second reviewer.

### Data extraction

One reviewer extracted relevant data on COS characteristics consistent with the study aims and primary outcomes of interest using a pre-designed extraction template. The primary outcomes were: COS target population, setting for application, stakeholders involved, initial information sources, consensus methods, outcomes included, PROMs recommended, and criteria and process for selecting PROMs. Setting for application was based on Lipscomb’s framework for cancer outcomes research [18], which posits three arenas for the application of PROs: micro (e.g., individual patient management and care), meso (e.g., comparative effectiveness research), and macro (e.g., population-level surveillance). Stakeholders referred to participants in the development process and included patients and the public, health care practitioners, regulators, industry representatives, and researchers. Initial information sources were the approaches used to identify possible outcomes for consideration, while consensus methods referred to the approaches used to elicit opinions and prioritise outcomes.

The verbatim names of PROs in the included COS were extracted and mapped to conceptual core areas and domains specified in the COMET taxonomy for the classification of outcomes in COS [19]. The COMET core areas are physiological/clinical, life impact, and resource use. Outcomes with similar names (defined as having at least one identical or synonymous word) were documented and consolidated. This process was performed by one reviewer and overseen and cross-checked for completeness by a second reviewer with methodological expertise in PRO research.

### Quality of reporting

The Core Outcome Set – STAndards for Reporting (COS-STAR) checklist [9] was used to examine the completeness, transparency, and accuracy of COS reporting in the included studies. The checklist contains 25 items considered essential for reporting on the development of a COS; structured around the introduction, methods, results, and discussion sections of a manuscript. For example, a checklist item for the study methods is to describe how the consensus process was undertaken [9]. Each study was assessed against the COS-STAR criteria to guide critical appraisal of the COS and facilitate comparison of their approaches. This provided insight into the extent to which the included studies aligned with published guidance for COS development.

### Synthesis of results

Results are presented in tabular format and using narrative synthesis.

## Results

**Fig. 1:**
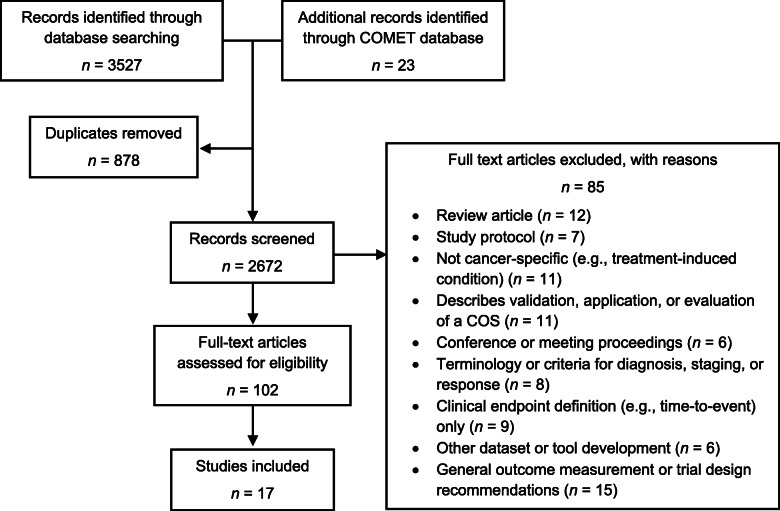
Identification and selection of studies

The searches returned 3527 citations and 23 additional records were identified from the COMET database. After the removal of 878 duplicates, 2672 titles and abstracts were screened for relevance. After title and abstract screening, 119 articles were retrieved for full-text examination. Of these, 85 were excluded with reasons cited in Fig. 1, and 17 were included in the review.

### Quality of reporting

The completeness and accuracy of COS reporting as assessed by the COS-STAR checklist was variable. Studies reported between 13 (52%) and 25 (100%) checklist items. Seven reported 85% of items or more, and 10 reported between 52% and 72%. The most comprehensive reporting was evident for two studies that used the COS-STAR checklist. Detailed information on the composition and characteristics of the participant sample was provided by only 5 studies. Gaps were also evident for reporting methods: only 4 studies followed a protocol, 6 studies did not explain which outcomes were dropped or introduced, 4 studies did not describe the outcome scoring process, and 5 studies did not explain how consensus was determined.

### Characteristics of COS

COS characteristics including intended setting, target population, information sources, consensus methods, stakeholders involved, and nature of PRO recommendations are reported in Table 2. The included COS were typically developed for use in clinical trials and most were specific to a cancer type (*n* = 14, 82%). The cancer types for which specific COS have been developed include breast (*n* = 2) [28, 32], lung (*n* = 1) [30], colorectal (*n* = 2) [29, 35], prostate (*n* = 4) [21, 23, 27, 31], ovarian (*n* = 1) [22], head and neck (*n* = 2) [20, 34], esophageal (*n* = 1) [25] and pancreatic (*n* = 1) [24]. Three COS contained outcomes relevant across cancer types [26, 33, 36]. Four studies focused on outcomes experienced immediately after surgery and the remainder were not specific to an intervention or treatment.

**Table 2:** Characteristics of the included COS studies

Author	Scope of core outcome set			Development methods		Stakeholder expertise			Nature of PRO recommendations
	Setting for COS use	Population	Intervention	Outcome identification	Consensus	Patient	Clinical	Other	
Avery [20]	Clinical effectiveness trials in esophageal cancer resection surgery	Adults with oesophageal cancer	Surgery	Patient interviews	Delphi questionnaire	Yes	Nurse	–	Outcomes only
				National audit of outcomes	Consensus meeting		Surgeon		
				Systematic review					
Chen [21]	Prostate cancer treatment trials	Men with prostate cancer	Not specified	Systematic review	Consensus meeting	Yes	Physician	Researcher	Suggests relevant measures
Chera [22]	Head & neck cancer treatment trials	Adults with head & neck cancer	Not specified	Literature review	Consensus meeting	Yes	Medical oncologist	HRQOL expert	Specific measures recommended
					Survey		Radiation oncologist	Psychologist	
							Surgeon		
Donovan [23]	Ovarian cancer treatment trials	Women with ovarian cancer	Not specified	Literature review	Consensus meeting	Yes	Gynecologic oncologist	–	Suggests relevant measures
							Medical oncologist		
							Nurse		
Geerse [24]	Not specified	Adult cancer survivors	Not specified	Based on the ICF	Delphi questionnaire	Yes	Nurse practitioner	Dietician	Specific measures recommended
							Oncology nurse	Social worker	
							Physician	Physical therapist	
								Psychologist	
Gerrtisen [25]	Nationwide prospective pancreatic cancer registry	Adults with pancreatic or periampullary cancer	Treated with curative intent or in palliative setting	Extracted PROMs from a systematic review and RCTs from the PROMOTION Registry	Delphi questionnaire	Yes	Gastroenterologist	Dietician	Outcomes only
							Medical oncologist		
							Nurse		
							Radiotherapist		
							Surgeon		
Howell [26]	Outcome measurement in routine cancer care	Adults with cancer	Not specified	Literature review	Delphi questionnaire	Yes	Oncologist	Administrator	Suggests relevant measures
					Consensus meeting		Physician	Dietician	
							Radiation oncologist	Psychologist	
								Social worker	
								Researcher	
Maclennan [27]	Localised prostate cancer effectiveness trials	Men with prostate cancer aged 45–75	Surgery	Systematic review	Delphi questionnaire	Yes	Cancer nurse specialist	–	Outcomes only
				Patient interviews	Consensus meeting		Oncologist		
							Surgeon		
Mak [28]	Routine clinical practice	Adults with newly diagnosed lung cancer	Any	Literature review	Delphi questionnaire	Yes	Medical oncologist	Registry expert	Specific measures recommended
					Teleconferences		Radiation oncologist	Researchers	
							Palliative care specialist		
							Pulmonologist		
							Specialist nurse		
							Surgeon		
Martin [29]	Routine clinical practice	Men with localised prostate cancer	Active monitoring, radiotherapy, surgery	Not specified	Delphi questionnaire	Yes	Radiation oncologist	Registry expert	Specific measures recommended
					Teleconferences		Urologist	Researchers	
McNair [30]	Clinical effectiveness trials in colorectal cancer surgery	Adults with colon or rectal cancer	Surgery	Systematic review	Delphi questionnaire	Yes	Clinical nurse specialist	Caregiver	Outcomes only
				Patient interviews	Consensus meeting		Surgeon		
				Analysis of patient information leaflets					
Morgans [31]	Routine clinical practice	Men with advanced prostate cancer	Any	Literature review	Delphi questionnaire	Yes	Medical oncologist	Epidemiologist	Specific measures recommended
					Teleconferences		Radiation oncologist		
							Oncology nurse		
							Palliative care specialist		
							Urologist		
Ong [9]	Routine clinical practice	Women with breast cancer	Not specified	Literature review	Delphi questionnaire	Yes	Nurse	Epidemiologist	Specific measures recommended
				Review of breast cancer registries	Videoconferences		Medical oncologist		
				Focus groups with patients			Radiation oncologist		
							Pathologist		
							Radiologist		
							Palliative care specialist		
							Surgeon		
Potter [32]	Research and audit studies in reconstructive breast surgery	Women with breast cancer	Surgery	Systematic review	Delphi questionnaire	Yes	Clinical nurse specialist	Industry representative	Outcomes only
				Interviews with patients and healthcare professionals	Consensus meeting		Surgeon		
								Psychologist	
Reeve [33]	Adult cancer treatment trials	Adults with cancer	Not specified	Systematic review	Teleconferences	Yes	Clinicians (not specified)	Industry representative	Outcomes only
				Analysis of primary data sources	Consensus meeting		Clinical trials cooperative group administration		
								Methodological expert	
								Researcher	
								Statistician	
Tschiesner [34]	Clinical studies & encounters	Adults with head & neck cancer	Not specified	Systematic review	Consensus conference	Yes	Nurse	Physiotherapist	Outcomes only
				Patient interviews			Otolaryngologist	Psychologist	
				Survey of health professionals	Nominal group technique		Medical oncologist	Social worker	
				Multi-centre study			Radiation oncologist		
							Surgeon		
Zerillo [29]	Value-based healthcare efforts in colorectal cancer care	Adults with colon or rectal cancer	Not specified	Literature review	Delphi questionnaire	Yes	Oncology nurse	Epidemiologist	Specific measures recommended
				Review of colorectal cancer registries	Videoconferences		Medical oncologist		
				Focus groups with patients					
							Radiation oncologist		
							Palliative care specialist		
							Surgeon		

### Development methods

The most common approach to generating an initial outcome list was carrying out a literature or systematic review, (*n* = 15, 88%). Of these studies, 8 also solicited the views of patients and/or health professionals via interviews or focus groups. Five studies referred to additional sources including registries [29, 32], primary data sources [36], a multi-centre study [20], patient leaflets [35], and a national audit of outcomes [35]. The most common method for prioritising outcomes was administering a Delphi questionnaire (*n* = 12, 71%). The second most common method was conducting a consensus meeting (*n* = 9, 53%), where stakeholders would meet face-to-face to discuss and agree on the final outcomes. Studies typically used a survey method followed by a discussion via consensus meeting, teleconference, or videoconference (*n* = 11, 65%), and all studies used at least one of these methods.

### Stakeholders

All studies obtained input from clinicians and patients in the development process. Three studies did not describe the profession or expertise of the clinicians involved. Among those that did, the most common clinical professions were surgeon (*n* = 7), radiation oncologist (*n* = 6), medical oncologist (*n* = 5), and clinical nurse specialist (*n* = 5). Allied healthcare professionals involved were psychologist (*n* = 5), dietician (*n* = 3), social worker (*n* = 3), and physical therapist (*n* = 1). Additional stakeholders included administrator, researcher, methodological expert, HRQOL expert, registry expert, epidemiologist, statistician, and industry representative.

### Recommended PROs

The COS included between 4 and 22 outcome domains, totaling 137 PROs across the included studies. After consolidating those that were similar or overlapping, 90 PROs remained for classification using the COMET taxonomy, presented in Table S2.

### Classification of PROs

Across the included COS there were 46 physiological/clinical PROs, 39 life impact PROs, and 5 resource use PROs. As expected, the breakdown of physiological/clinical domains reflected the profile of cancer types for which COS were developed. The most common physiological classifications were ‘renal and urinary’ (9 outcomes), ‘ear/labyrinth’ (8 outcomes), ‘gastrointestinal’ (6 outcomes), and ‘general’ (9 outcomes), which refers to outcomes affecting the whole body that cannot be attributed to one system. Life impact PROs were classified within the domains of ‘physical functioning’ (10 outcomes), ‘cognitive functioning’ (3 outcomes), ‘emotional functioning’ (12 outcomes), ‘role functioning’ (1 outcome), ‘social functioning’ (9 outcomes), ‘global quality of life’ (1 outcome), ‘delivery of care’ (2 outcomes), and ‘personal circumstances’ (2 outcomes). All studies included a measure of life impact and 94% (16 COS) included at least one physiological outcome. Nearly all (16 COS) included an outcome from the physical functioning domain, 65% (11 COS) included an outcome from the emotional functioning domain, 47% (8 COS) included an outcome from the social functioning domain, 29% (5 COS) included an outcome from the cognitive functioning domain, and 6% (1 COS) included an outcome from the role functioning domain. Only 18% (3 COS) included a resource use outcome. The most common outcomes were physical function (9 COS), sexual (dys) function (9 COS), pain (9 COS), fatigue (8 COS), emotional function (8 COS), global quality of life (7 COS), bowel symptoms (5 COS), and social function (5 COS).

### Approaches to identifying and selecting recommended PROMs

Most COS (59%) did not recommend how to assess the core PRO domains. Seven studies (47%) did not provide any measurement guidance, of which three mentioned that instrument selection was part of planned future research. Three studies (12%) provided general instrument guidance that was not based on a formal process of identifying and evaluating measures [22, 31, 34]. Of these, two studies suggested possible measures by providing a list of relevant PROMs mapped to the recommended COS domains, but they did not preference any specific measure(s) [31, 34]. The other study cited the two most commonly used cancer PROMs for the assessment of multidimensional HRQOL, each with cancer-specific modules [22].

The seven studies that recommended specific PROMs varied in their approaches to instrument selection. Two studies conducted reviews of PROMs and recommended measures that aligned with the review criteria [26, 33], with one linking individual items from three identified PROMs to the agreed-upon core outcomes [26]. Four studies evaluated measures based on the coverage of included domains and alignment with the International Society for Quality of Life Research (ISOQOL) research standards, which specify psychometric quality, clinical interpretability, and feasibility of implementation in daily practice [27, 29, 30, 32]. One study did not describe its process for selecting PROMs [21].

The most commonly recommended measure was the European Organisation for Research and Treatment of Cancer (EORTC) Quality of Life - Core questionnaire (QLQ-C30) (*n* = 5). Disease-specific modules were recommended alongside the QLQ-C30 for head and neck cancer (HN35), breast cancer (BR23), colorectal cancer (CR29), ovarian cancer (OV28), and lung cancer (LC13). One COS recommended a single item from the liver metastases module (LMC21) and another recommended two items from the prostate cancer module (PR25). The Functional Assessment of Cancer Therapy - General (FACT-G) and its ovarian cancer module (FACT-O) were a recommended alternative to the QLQ-C30 and OV28. The Expanded Prostate Cancer Index Composite (EPIC-26) was recommended twice and the Impact of Cancer questionnaire (IOCv2), Quality of Life in Adult Cancer Survivors questionnaire (QLACS), Distress Thermometer and Problem List (DT/PL), Memorial Sloan Kettering Cancer Centre (MSKCC) Bowel Function Instrument, University of Washington Quality of Life Questionnaire (UW-QOL), and endocrine symptoms subscale of the FACT were all recommended once.

## Discussion

This systematic review provides a detailed overview of consensus-based COS containing PROs that have been developed for use with cancer populations. There is growing interest in standardising outcome measurement in health research, which has resulted in an extensive number of COS, elements within them, and parallel initiatives to support robust COS development [1, 9, 11, 17]. By examining what COS are available for use in research with cancer populations, this review aimed to identify where further guidance is needed for COS developers using consensus-based approaches, particularly with regard to the selection of PROs and PROMs. The included studies typically developed COS using comprehensive and stepwise processes, often drawing on published literature as an initial information source, then eliciting an expert consensus opinion from different stakeholder groups including patients and clinicians. While development methods were similar across studies, approaches to identifying, selecting, and recommending PROMs varied substantially and nearly half of the studies did not address how to measure core outcomes. Most COS were intended for use in clinical trials and therefore focused on assessing acute treatment-related toxicities and symptoms over long-term psychological and social outcomes. One COS was specifically aimed at health-related problems in adult cancer survivors [26], while the four core sets for breast, lung, prostate, and colorectal cancers developed by the International Consortium for Health Outcomes Measurement (ICHOM) sought to incorporate outcomes from diagnosis to treatment completion and long-term survivorship [21, 29, 30, 32]. Most COS focused on a specific cancer. The National Cancer Institute developed three core sets of disease-specific symptoms to measure in trials for head and neck, prostate, and ovarian cancer [22, 31, 34], in addition to a core set of symptoms applicable in all cancer treatment trials [36]. Since clinical trials are increasingly being designed to capture the long-term effects of cancer treatment on HRQOL, COS specifically targeting long-term survivorship PROs would be of value for longitudinal studies and surveillance [13].

Reviews have found that synthesising evidence from PRO studies can be challenging because of the various PROMs used and subsequent differences in outcome definitions, and how the measures are administered, interpreted, and scored [13]. COS have been proposed as a way of addressing problems associated with outcome inconsistency in research [3]. However, the review findings suggest that efforts to standardise outcome assessment in cancer through the development of COS may be undermined by a lack of recommendations on how to measure PROs included in COS. Specifically, it was found that COS studies that did recommend measures to assess core PROs employed different selection criteria and processes that were often not well described or justified. Meanwhile, COS studies that did not recommend measures to assess core PROs often neglected to adequately define and describe the agreed-upon outcomes or specify whether they should be assessed via unidimensional or multidimensional measures and/or subscales. As a result, outcomes may be interpreted, operationalised, and measured differently by COS users and the compromising effect of outcome inconsistency on research quality and efficiency is unlikely to be resolved. Previous content examination of PROMs has found that the component questions of instruments with similar names do not always address comparable issues [3].

The provision of well-informed guidance on how to measure PROs in a COS, which was absent from most of the included studies, is critical for COS usefulness and adoption. The COSMIN/COMET guideline provides a practical four-step method to guide COS developers in undertaking this process, including the following recommendations concerning the selection of measures for a COS: 1) select only one instrument for each outcome, 2) ensure there is at least high-quality evidence for good content validity, internal consistency, and feasibility of the instrument(s), and 3) obtain consensus on the instrument(s) [11]. Uptake of the COSMIN/COMET guideline by future COS developers will help to ensure that core outcomes can be operationalised and measured consistently. Involving stakeholders with methodological and psychometric expertise in the COS development process may facilitate adherence to the guideline and selection of appropriate PROMs. To further prompt COS developers to recommend measures, existing standards for COS study design and reporting could be expanded to include items relevant to determining how to measure core outcomes (i.e., defining a core outcome *measurement* set) and reporting on this process. A comprehensive approach to researching and evaluating appropriate PROMs was demonstrated by the ICHOM initiatives, which selected measures on the basis of outcome coverage, psychometric quality, clinical interpretability, and feasibility to assess and implement in daily practice [21, 29, 30, 32]. This assessment was based in part on minimum standards for PROMs recommended by ISOQOL, another useful resource for COS developers intending to include PROs [37].

An alternative approach to addressing PRO measurement problems has been to create common metrics and crosswalk between instruments [38]. The Patient-Reported Outcomes Measurement Information System (PROMIS) is an item-response theory calibrated metric that was developed to assess domains (i.e., item banks) relevant across health conditions while addressing concerns about the precision, standardisation, quality, and comparability of PROMs [39]. The PROMIS item banks can be customised and flexibly administered while remaining directly comparable [38]. Similarly, the EORTC has used item-response theory to create item banks for all symptom and functional domains of the QLQ-C30, which is one of the most commonly used PROMs in cancer trials [40]. Scores from any subset of items within an item bank are calibrated on the same common metric and therefore directly comparable, enabling dynamic and individualised assessment utilising computer adaptive testing as well as the generation of short forms [40]. Common metrics allow similar instruments to be linked and rescored on the metric (referred to as crosswalk or mapping) [41]. A growing number of studies have developed and applied methods to link scores from common metrics with those from other similar PROMs; enabling meaningful comparison of outcomes across studies [42, 43]. However, mapping or crosswalk is not always accurate or appropriate [43] and the strength of this approach depends on the degree of overlap between measures [44]. Mapped estimates increase uncertainty and thus administering a common metric (i.e., preference-weighted measure) in the first place is considered preferable [43, 44]. Given their shared objective of improving outcome standardisation, an exploration of how COS can complement efforts to create common metrics, item banks, and crosswalk between instruments is an avenue for future investigation.

The insights of this review into the identification and selection of PROMs for COS have highlighted shortcomings, possible directions and areas where further guidance for COS developers may be required in the literature. Strengths of this review include a comprehensive search strategy targeting the COMET database of completed and ongoing COS studies as well as academic databases, which ensured that as many relevant studies as possible were identified. Another strength was the application of two recently developed COS resources; the COS-STAR checklist was used to formally assess the completeness and accuracy of COS reporting across the included studies [9], and the COMET taxonomy was used to classify outcomes [19]. Our stringent inclusion criteria designed to address the review objectives meant that we did not exhaustively review all studies that could be considered a COS, which could be a limiting factor.

## Conclusions

Core outcome sets have the potential to resolve issues associated with outcome inconsistency in health research, if they deliver clear and actionable recommendations. This review found that some cancer-related COS may be undermined by a lack of recommendations on how to measure core PROs, highlighting the need for further emphasis on this component of COS development. Most cancer-related COS did not undertake this step, and methods for identifying, appraising, and selecting appropriate PROMs varied substantially among those that did. Selecting PROMs as a part of COS development, with the use of resources designed to support this process, will optimise the usefulness of COS and facilitate their consistent implementation. Although their adoption may not resolve all aspects of outcome inconsistency, well-developed COS promote research that is more efficient and informative for decision-making by regulators, health care providers, and patients [14].

## Supplementary information

**Additional file 1: Table S1.** PRISMA 2009 checklist.

**Additional file 2: Table S2.** Classification of patient-reported outcomes included in cancer-related core outcome sets.

## Data Availability

Not applicable.

## Abbreviations

COMET: Core Outcome Measures in Effectiveness Trials
COS: Core outcome set(s)
COS-STAD: Core Outcome Set-Standards for Design
COS-STAP: Core Outcome Set-Standardised Protocol Items
COS-STAR: Core Outcome Set-Standards for Reporting
COSMIN: Consensus-based Standards for the Selection of Measurement Instruments
EORTC: European Organisation for Research and Treatment of Cancer
HRQOL: Health-related quality of life
ICHOM: International Consortium for Health Outcomes Measurement
ISOQOL: International Society for Quality of Life Research
OMERACT: Outcome Measures in Rheumatology
PRISMA: Preferred Reporting Items of Systematic Reviews and Meta-Analyses
PRO(s): Patient-reported outcome(s)
PROM(s): Patient-reported outcome measure(s)
PROMIS: Patient-Reported Outcomes Measurement Information System
PROSPERO: International Prospective Register of Systematic Reviews
QLQ-C30: Quality of Life-Core questionnaire
