# Supplementary material for: Core outcome sets in cancer and their approaches to identifying and selecting patient-reported outcome measures: a systematic review
Source: J Patient Rep Outcomes. 2020 Sep 15;4:77. doi: 10.1186/s41687-020-00244-3 (PMC7492323; doi:10.1186/s41687-020-00244-3)
Supplement: Supplementary file 2 — Additional file 2: Table S2. Classification of patient-reported outcomes included in cancer-related core outcome sets. [file 41687_2020_244_MOESM2_ESM.docx]

Table S2. Classification of patient-reported outcomes included in cancer-related core outcome sets

| **Core area** | | **Outcome** | **Alternative name** | **# of COS** | **All cancers** | **Breast** | **Colorectal** | **Esophageal** | **Head & neck** | **Lung** | **Ovarian** | **Prostate** | **Pancreatic** |
| --- | --- | --- | --- | --- | --- | --- | --- | --- | --- | --- | --- | --- | --- |
| Physiological/clinical | |  |  | 16 | ✓ | ✓ | ✓ | ✓ | ✓ | ✓ | ✓ | ✓ | ✓ |
|  | Ear & labyrinth | Voice hoarseness | - | 1 |  |  |  |  | ✓ |  |  |  |  |
|  |  | Oral pain | - | 1 |  |  |  |  | ✓ |  |  |  |  |
|  |  | Dry mouth | - | 1 |  |  |  |  | ✓ |  |  |  |  |
|  |  | Dental health | - | 1 |  |  |  |  | ✓ |  |  |  |  |
|  |  | Opening mouth/trismus | - | 1 |  |  |  |  | ✓ |  |  |  |  |
|  |  | Swallow | - | 1 |  |  |  |  | ✓ |  |  |  |  |
|  |  | Taste | - | 1 |  |  |  |  | ✓ |  |  |  |  |
|  |  | Excess/thick mucus/saliva | - | 1 |  |  |  |  | ✓ |  |  |  |  |
|  | Endocrine | Hormonal symptoms | Side effects of hormonal therapy | 4 |  |  |  |  |  |  |  | ✓ |  |
|  |  | Vasomotor symptoms | - | 1 |  | ✓ |  |  |  |  |  |  |  |
|  | Gastrointestinal | Gastrointestinal symptoms | - | 1 |  |  | ✓ |  |  |  |  |  |  |
|  |  | Indigestion | Problems with acid indigestion and heartburn | 2 |  |  |  | ✓ |  |  | ✓ |  |  |
|  |  | Abdominal pain | - | 1 |  |  |  |  |  |  | ✓ |  |  |
|  |  | Bloating | - | 1 |  |  |  |  |  |  | ✓ |  |  |
|  |  | Cramping | - | 1 |  |  |  |  |  |  | ✓ |  |  |
|  |  | Presence of stoma | - | 1 |  |  | ✓ |  |  |  |  |  |  |
|  | General | Pain | Sensation of pain | 9 | ✓ | ✓ | ✓ |  | ✓ | ✓ |  | ✓ |  |
|  |  | Fatigue | - | 8 | ✓ | ✓ | ✓ |  |  | ✓ |  | ✓ | ✓ |
|  |  | Nausea/Vomiting | Nausea  Vomiting | 3 | ✓ |  |  |  |  |  | ✓ |  |  |
|  |  | Arthralgia | - | 1 |  | ✓ |  |  |  |  |  |  |  |
|  |  | Anorexia (appetite problems) | Appetite | 2 | ✓ |  |  |  |  |  |  |  |  |
|  |  | Weight changes | Weight gain  Weight loss | 2 |  |  |  |  |  |  | ✓ | ✓ |  |
|  |  | Sleep/wake function disturbance | - | 1 | ✓ |  |  |  |  |  |  |  |  |
|  |  | Insomnia | - | 2 | ✓ | ✓ |  |  |  |  |  |  |  |
|  |  | Overall symptom experience | - | 1 | ✓ |  |  |  |  |  |  |  |  |
|  | Metabolism & nutrition | Nutritional status | Severe nutritional problems  Dietary issues | 3 | ✓ |  | ✓ | ✓ |  |  |  |  |  |
|  | Musculoskeletal & connective tissue | Shoulder disability/motion | - | 1 |  |  |  |  | ✓ |  |  |  |  |
|  |  | Arm symptoms | - | 1 |  | ✓ |  |  |  |  |  |  |  |
|  | Nervous system | Neuropathy | Peripheral neuropathy  Sensory neuropathy | 3 | ✓ | ✓ | ✓ |  |  |  |  |  |  |
|  | Psychiatric | Depression | - | 4 | ✓ | ✓ | ✓ |  |  |  |  |  |  |
|  |  | Anxiety | - | 3 | ✓ | ✓ |  |  |  |  |  |  |  |
|  | Renal & urinary | Urinary incontinence | Stress urinary incontinence | 3 |  |  |  |  |  |  |  | ✓ |  |
|  |  | Urinary irritation/obstruction | Urinary obstruction  Urinary irritation  Urinary obstruction/irritation | 2 |  |  |  |  |  |  |  | ✓ |  |
|  |  | Urinary symptoms | Urinary function | 1 |  |  |  |  |  |  |  | ✓ |  |
|  |  | Bowel symptoms | Bowel function  Bowel-related symptoms | 5 |  |  | ✓ |  |  |  |  | ✓ |  |
|  |  | Fecal incontinence | Fecal urgency  Fecal leakage | 3 |  |  | ✓ |  |  |  |  | ✓ |  |
|  |  | Defecation | - | 1 |  |  |  |  |  |  |  |  | ✓ |
|  |  | Stool frequency | - | 1 |  |  | ✓ |  |  |  |  |  |  |
|  |  | Constipation | - | 1 | ✓ |  |  |  |  |  |  |  |  |
|  |  | Diarrhoea | - | 2 | ✓ |  | ✓ |  |  |  |  |  |  |
|  | Reproductive system & breast | Satisfaction with breast | - | 1 |  | ✓ |  |  |  |  |  |  |  |
|  |  | Breast symptoms | - | 1 |  | ✓ |  |  |  |  |  |  |  |
|  |  | Vaginal symptoms | - | 2 |  | ✓ | ✓ |  |  |  |  |  |  |
|  |  | Dyspnoea | - | 3 | ✓ |  |  |  |  | ✓ |  |  |  |
|  |  | Cough | - | 1 |  |  |  |  |  | ✓ |  |  |  |
|  | Subcutaneous tissue & skin | Skin changes | - | 1 |  |  |  |  | ✓ |  |  |  |  |
| Life impact | |  |  | 17 | ✓ | ✓ | ✓ | ✓ | ✓ | ✓ | ✓ | ✓ | ✓ |
|  | Physical functioning | Physical function | Physical wellbeing  Physical ability  Functional domain | 9 |  | ✓ | ✓ |  | ✓ | ✓ |  | ✓ | ✓ |
|  |  | Mobility | Perceived mobility  Objective mobility | 2 | ✓ |  | ✓ |  |  |  |  |  |  |
|  |  | Instrumental activities of daily living | Ability to do work/usual activities  Carrying out daily routine | 3 | ✓ |  |  |  | ✓ |  |  |  | ✓ |
|  |  | Driving | - | 1 | ✓ |  |  |  |  |  |  |  |  |
|  |  | The ability to eat/drink | Eating  Drinking | 3 | ✓ |  |  | ✓ | ✓ |  |  |  |  |
|  |  | Reading | - | 1 | ✓ |  |  |  |  |  |  |  |  |
|  |  | Looking after one’s health | - | 1 | ✓ |  |  |  |  |  |  |  |  |
|  |  | Energy & drive functions | - | 2 | ✓ |  |  |  | ✓ |  |  |  |  |
|  |  | Sexual function | Sexual dysfunction  Sexual functions  Sexual symptoms | 9 | ✓ | ✓ | ✓ |  |  |  | ✓ | ✓ |  |
|  |  | Erectile dysfunction | - | 1 |  |  | ✓ |  |  |  |  |  |  |
|  | Cognitive functioning | Cognitive function | Cognitive problems | 4 | ✓ | ✓ |  |  |  | ✓ |  |  |  |
|  |  | Attention functions | - | 1 |  |  |  |  |  |  |  |  |  |
|  |  | Making decisions | - | 1 | ✓ |  |  |  |  |  |  |  |  |
|  | Emotional functioning | Emotional function | Emotional functions  Emotional wellbeing  Mental wellbeing | 8 | ✓ | ✓ | ✓ |  | ✓ | ✓ |  | ✓ |  |
|  |  | Fear of recurrence | - | 2 |  |  |  |  |  |  | ✓ |  | ✓ |
|  |  | Negative feelings | - | 1 |  |  |  |  |  |  |  |  | ✓ |
|  |  | Coping | - | 2 | ✓ |  |  |  |  |  |  |  | ✓ |
|  |  | Handling stress & other psychological demands | - | 1 | ✓ |  |  |  |  |  |  |  |  |
|  |  | Psychological adjustment overall | - | 1 | ✓ |  |  |  |  |  |  |  |  |
|  |  | Body image | Self-concept/body image | 2 | ✓ | ✓ |  |  |  |  |  |  |  |
|  |  | Self-esteem | - | 1 |  | ✓ |  |  |  |  |  |  |  |
|  |  | Normality | - | 1 |  | ✓ |  |  |  |  |  |  |  |
|  |  | Cosmetic satisfaction | - | 1 |  | ✓ |  |  |  |  |  |  |  |
|  |  | Meaning & spirituality | - | 1 | ✓ |  |  |  |  |  |  |  |  |
|  |  | Subjective wellbeing | - | 1 | ✓ |  |  |  |  |  |  |  |  |
|  | Role functioning | Ability to work | - | 1 |  | ✓ |  |  |  |  |  |  |  |
|  | Social functioning | Social function | Social domain | 5 | ✓ | ✓ | ✓ |  | ✓ | ✓ |  |  |  |
|  |  | Family relationships | Relationships with partner/family | 2 |  |  |  |  | ✓ |  |  |  | ✓ |
|  |  | Basic interpersonal interactions | - | 1 | ✓ |  |  |  |  |  |  |  |  |
|  |  | Complex interpersonal interactions | - | 1 | ✓ |  |  |  |  |  |  |  |  |
|  |  | Social support | - | 1 | ✓ |  |  |  |  |  |  |  |  |
|  |  | Immediate family | - | 2 | ✓ |  |  |  | ✓ |  |  |  |  |
|  |  | Friends | - | 1 | ✓ |  |  |  |  |  |  |  |  |
|  |  | Health professionals | - | 2 | ✓ |  |  |  |  |  |  |  |  |
|  |  | Individual attitudes of immediate family members | - | 1 | ✓ |  |  |  |  |  |  |  |  |
|  | Global quality of life | Global QOL | Global HRQOL  QOL  HRQOL  General QOL  Global health status/QOL  General health  Overall wellbeing | 7 | ✓ | ✓ | ✓ | ✓ |  | ✓ |  | ✓ | ✓ |
|  | Delivery of care | Satisfaction with caregivers | - | 1 |  |  |  |  |  |  |  |  | ✓ |
|  |  | Satisfaction with services  & organisations of care | - | 1 |  |  |  |  |  |  |  |  | ✓ |
|  | Personal circumstances | Economic self-sufficiency | - | 2 | ✓ |  |  |  | ✓ |  |  |  |  |
|  |  | Financial impact | - | 2 |  | ✓ |  |  | ✓ |  |  |  |  |
| Resource use | |  |  |  |  |  |  |  |  |  |  |  |  |
|  | Economic | Social security series, systems & policies | - | 1 | ✓ |  |  |  |  |  |  |  |  |
|  |  | Health services, systems & policies | - | 1 | ✓ |  |  |  |  |  |  |  |  |
|  | Need for further intervention | Medication use | - | 1 |  |  |  |  |  |  |  |  | ✓ |
|  |  | Enzyme replacement therapy use | - | 1 |  |  |  |  |  |  |  |  | ✓ |
|  |  | Products and substances for consumption | - | 1 |  |  |  |  | ✓ |  |  |  |  |
